# Effects of brain–computer interface output modalities on motor recovery after stroke: a systematic review and network meta-analysis

**DOI:** 10.3389/fnins.2026.1978309

**Published:** 2026-10-27

**Authors:** Haoying Jiang, Xinzhao Zhang, Jingying Xu, Yuyao Fu, Na Jin, Yao Xiao, Lili Fang

**Affiliations:** 1 964th Hospital of the Joint Logistic Support Force, People's Liberation Army General Hospital, Changchun, China; 2 The Chinese University of Hong Kong, Hong Kong, Hong Kong SAR, China

**Keywords:** brain-computer interface, functional electrical stimulation, motor recovery, robotic rehabilitation, stroke

## Abstract

**Background:**

The relative effects of different brain-computer interface output modalities for post-stroke motor rehabilitation remain unclear.

**Objectives:**

To synthesize randomized evidence on brain–computer interface-assisted motor rehabilitation after stroke, with emphasis on functional electrical stimulation-triggered and robot-assisted output modalities and the certainty of evidence.

**Methods:**

Sixteen randomized controlled trials published from January 2020 to October 2025 were identified from PubMed, Web of Science, Embase, IEEE Xplore, the Cochrane Library, and MEDLINE. Interventions included brain-computer interface-triggered functional electrical stimulation, brain-computer interface-controlled robotic rehabilitation, and conventional rehabilitation. The primary outcome was motor recovery measured by the Fugl-Meyer Assessment. A frequentist random-effects network meta-analysis was performed, and certainty of evidence was assessed using CINeMA.

**Results:**

No trial directly compared the two active brain-computer interface modalities, resulting in a star-shaped network. Compared with conventional rehabilitation, brain-computer interface-triggered functional electrical stimulation improved motor recovery (standardized mean difference 0.57, 95% confidence interval 0.12 to 1.01), whereas brain-computer interface-controlled robotic rehabilitation showed an uncertain effect (0.17, −0.25 to 0.60). The indirect comparison numerically favored brain-computer interface-triggered functional electrical stimulation over brain-computer interface-controlled robotic rehabilitation, but the confidence interval crossed the null (0.39, −0.22 to 1.01). Certainty was limited by indirectness, imprecision, and heterogeneity.

**Conclusion:**

Brain–computer interface-triggered functional electrical stimulation showed a clearer signal of benefit vs. conventional rehabilitation than brain-computer interface-controlled robotic. However, the comparison between the two active modalities relies entirely on indirect evidence from a star-shaped network, has very low confidence, and does not establish comparative superiority. P-score rankings are exploratory pending direct head-to-head trials.

## Introduction

Stroke remains a leading cause of mortality and chronic disability worldwide, with steadily increasing burden (Feigin et al., 2025; GBD 2021 Stroke Risk Factor Collaborators, 2024). Many survivors live with persistent deficits that affect mobility, self-care, and daily participation. Among these sequelae, motor impairment is one of the most prevalent and clinically important, directly constraining functional independence and posing a major rehabilitation challenge (Langhorne et al., 2011). Accordingly, improving motor recovery constitutes a cornerstone of post-stroke care. For rehabilitation clinicians, the central question is not only whether a technology can induce neurophysiological change, but whether it can produce measurable gains in motor performance, daily activity, and longer-term recovery.

Contemporary stroke rehabilitation is grounded in the view that recovery is shaped by activity-dependent neuroplasticity. Repetitive, task-oriented sensorimotor practice can reorganize residual neural networks and support functional improvement (Krakauer et al., 2012). This perspective has shifted attention toward interventions that require active engagement and provide feedback that is temporally linked to the patient's motor intention. In the subacute and chronic stages of stroke, pharmacological approaches for restoring motor function are scarce, making rehabilitation the principal avenue to clinically meaningful recovery (Langhorne et al., 2011; Krakauer et al., 2012).

Against this background, brain-computer interface (BCI)-assisted rehabilitation has emerged as a promising therapeutic strategy. BCIs decode brain activity into external commands, thereby coupling motor intention with sensory or motor output (Daly and Wolpaw, 2008). In stroke rehabilitation, most clinical strategies rely on non-invasive electroencephalography, which is safe, practical, and well-suited to capturing the temporal dynamics of motor intention (Abiri et al., 2019). Upon detection of motor-related signals followed by instant feedback, a closed-loop sensorimotor circuit is established. Such contingency may reinforce residual motor pathways and facilitate use-dependent plasticity (Mrachacz-Kersting et al., 2016).

Among the available output modalities for BCI-assisted stroke rehabilitation, functional electrical stimulation and robotic assistance have been most frequently evaluated in randomized trials. In BCI-FES systems, intention-related cortical activity triggers electrical stimulation of the affected muscles, aiming to re-establish the link between motor command and peripheral sensory input (Biasiucci et al., 2018). In BCI-Robot systems, decoded signals initiate or modulate robotic assistance, enabling structured and intensive movement practice under mechanical guidance (Ang et al., 2015). Both approaches operate within a closed-loop framework but engage the motor system differently. BCI-FES acts more directly on muscle activation and intention-driven sensorimotor coupling, whereas BCI-Robot emphasizes assisted movement execution and repeated task practice. These differences may influence both the magnitude and the clinical interpretation of treatment effects.

Over the past decade, evidence supporting BCI-based post-stroke rehabilitation has accumulated steadily. Several systematic reviews and meta-analyses have reported favorable effects, especially for upper-limb motor outcomes (Bai et al., 2020; Baniqued et al., 2021; Cervera et al., 2018; Mansour et al., 2022). Concurrently, the literature has grown increasingly heterogeneous. Trials differ in patient characteristics, signal acquisition methods, feedback modalities, treatment dose, control conditions and follow-up duration, and many have small sample sizes (Bai et al., 2020; Baniqued et al., 2021; Cervera et al., 2018; Mansour et al., 2022). A pooled effect for BCI-assisted therapy therefore does not answer whether the available trials support distinct clinical-effect estimates for BCI-triggered FES and BCI-controlled robotic assistance.

This knowledge gap has practical importance. BCI-FES and BCI-Robot are often subsumed under the same umbrella of BCI-based rehabilitation, yet they differ in therapeutic rationale, equipment requirements, and implementation pathways. These distinctions are not merely technical; they may influence which patients are most likely to benefit, which motor goals are targeted, and how the intervention can be implemented in routine rehabilitation. Pairwise modality-vs.-control estimates do not, by themselves, summarize the indirect BCI-FES-vs.-BCI-Robot contrast through a shared comparator (Bai et al., 2020; Mansour et al., 2022). As a result, the evidence base does not yet clearly indicate how these output modalities should be selected for different rehabilitation goals.

To address this limitation, we performed a systematic review and network meta-analysis of randomized controlled trials evaluating BCI-FES and BCI-Robot for post-stroke motor and functional recovery. Network meta-analysis organizes these clinical comparisons and their uncertainty, but it cannot isolate the causal contribution of the physical output modality or establish a mechanistic difference (Salanti, 2012). This review was designed to map the current randomized evidence for BCI output modalities in stroke rehabilitation, estimate their effects on motor recovery, and assess the certainty of the available evidence. Because no direct head-to-head trials were available, the aim was not to establish definitive superiority between modalities, but to clarify what can and cannot be inferred from the existing evidence network.

## Methods

### Study design and registration

This study was a systematic review and frequentist network meta-analysis comparing BCI-FES, BCI-Robot, and conventional rehabilitation for motor and functional recovery after stroke. The review was conducted in accordance with the Preferred Reporting Items for Systematic Reviews and Meta-Analyses extension for Network Meta-Analyses (PRISMA-NMA) (Hutton et al., 2015) and the Cochrane Handbook for Systematic Reviews of Interventions (Higgins et al., 2019). The protocol was registered in PROSPERO (CRD420251179410).

### Eligibility criteria

Studies were selected according to predefined criteria under the PICOS framework. Eligible participants were aged 18 years or older with ischemic or hemorrhagic stroke at any stage of recovery. The intervention nodes were BCI-FES or BCI-Robot. Nodes were defined according to the primary output modality that delivered intention-contingent feedback. Trials were classified as BCI-FES when decoded brain activity triggered peripheral electrical stimulation, and as BCI-Robot when decoded activity initiated or controlled robotic or exoskeleton-assisted movement. The comparator node was conventional rehabilitation without BCI integration, including routine physical therapy, occupational therapy, or standard multidisciplinary rehabilitation. The primary outcome was motor recovery measured by validated scales. The Fugl-Meyer Assessment for the upper or lower extremity was prioritized because it is widely used in stroke rehabilitation trials and allows comparison across studies. Secondary outcomes included broader functional measures such as balance, gait, walking ability, and activities of daily living when available. We included only randomized controlled trials published as full-text articles in peer-reviewed journals. Non-randomized studies, case reports, conference abstracts without full text, review articles, protocols, and animal studies were excluded.

### Literature search

We searched PubMed, Web of Science, Embase, IEEE Xplore, the Cochrane Library, and MEDLINE for studies published from January 2020 to October 2025. This period was chosen to focus on recent clinical applications as BCI technology continued to mature. The search strategy combined controlled vocabulary and free-text terms related to brain-computer interface, BCI, functional electrical stimulation, FES, robotic rehabilitation, stroke, and randomized controlled trial. Reference lists of relevant reviews and eligible studies were also screened to identify additional reports.

### Study selection and data extraction

Two reviewers independently screened titles and abstracts, then assessed the full texts of potentially eligible studies. Disagreements were resolved by discussion, with consultation of a third reviewer when necessary. For each included study, we extracted first author, publication year, sample size, participant characteristics, stroke stage, intervention details, comparator details, treatment dose, outcome measures, and post-intervention data (mean, standard deviation, and sample size). When change-from-baseline data were unavailable, post-intervention values were used. When multiple time points were reported, the end-of-treatment assessment was selected for the main analysis.

### Risk of bias assessment

Methodological quality was assessed independently by two reviewers using the original Cochrane Risk of Bias tool (Higgins et al., 2011). Seven domains were evaluated: random sequence generation, allocation concealment, blinding of participants and personnel, blinding of outcome assessment, incomplete outcome data, selective reporting, and other potential sources of bias. Each domain was rated as low, unclear, or high risk of bias. Disagreements were resolved through discussion.

### Statistical analysis

Continuous outcomes were summarized as standardized mean differences (SMDs) with 95% confidence intervals (CIs). SMDs were used because the included studies reported motor and functional outcomes on different scales. Review Manager 5.4 (RevMan 5.4) was used to organize pairwise data, prepare study-level summaries, and generate the risk-of-bias figures. The network meta-analysis was performed in R using a frequentist random-effects consistency model (Rücker, 2012; Rücker and Schwarzer, 2015). The main analyses and visualizations were implemented with the netmeta framework, and additional data handling and sensitivity procedures were completed in R. Three treatment nodes were defined in the main network: BCI-FES, BCI-Robot, and conventional rehabilitation. Because all direct comparisons used conventional rehabilitation as the shared comparator, the resulting network was star-shaped and contained no closed loops. Network plots were generated with node size proportional to the number of participants and edge thickness proportional to the number of studies.

### Assessment of transitivity, heterogeneity, and consistency

The transitivity assumption was assessed qualitatively by comparing key clinical and methodological characteristics across treatment comparisons, including stroke stage, target body region, and treatment dose. Statistical heterogeneity was quantified using Cochran's Q, the I^2^ statistic, and between-study variance (tau^2^) (Higgins et al., 2011). I^2^ values of 25%, 50%, and 75% were interpreted as low, moderate, and high heterogeneity, respectively. Formal tests for inconsistency, such as node-splitting or design-by-treatment interaction models, were not applicable because the network contained no closed loops. All network estimates were therefore obtained under the consistency assumption, which could not be empirically verified in this network. The absence of a test result should not be interpreted as evidence of consistency.

### Ranking, subgroup, sensitivity, and small-study effect analyses

Treatment ranking was summarized with P-scores, rankings were interpreted as exploratory because the network contained no closed loops and the active-vs.-active comparison was based entirely on indirect evidence, with higher values indicating a greater probability of superiority over competing interventions (Rücker and Schwarzer, 2015). Rankograms were also generated. In addition to the overall network, separate analyses were performed for Fugl-Meyer Assessment-Upper Extremity (FMA-UE) and Fugl-Meyer Assessment-Lower Extremity (FMA-LE) when sufficient data were available. Prespecified subgroup analyses were conducted according to stroke stage (subacute or chronic). Leave-one-out sensitivity analyses were performed separately for the direct comparisons of BCI-FES vs. conventional rehabilitation and BCI-Robot vs. conventional rehabilitation to examine the influence of individual studies on pooled estimates. Small-study effects and possible publication bias were explored visually using a comparison-adjusted funnel plot for the whole network and funnel plots stratified by treatment comparison (Chaimani and Salanti, 2012).

### Certainty of evidence

Certainty of evidence was assessed with the Confidence in Network Meta-Analysis (CINeMA) framework, which is grounded in GRADE methodology (Guyatt et al., 2008; Nikolakopoulou et al., 2020). Confidence in each network estimate was judged across six domains: within-study bias, reporting bias, indirectness, imprecision, heterogeneity, and incoherence. Final ratings were categorized as high, moderate, low, or very low. Detailed judgments are presented in Supplementary Table S1.

## Results

### Study selection, characteristics, and network structure

The study selection process is illustrated in Figure 1. Sixteen randomized controlled trials were included in the final analysis (Chen et al., 2020; Fu et al., 2023; Guo et al., 2022; Hu et al., 2021; Lee et al., 2022; Li et al., 2021, 2022; Liu et al., 2023; Luo, 2024; Ma et al., 2023; Miao et al., 2020; Ming et al., 2025; Wang et al., 2024; Yuan et al., 2021; Zanona et al., 2023; Zhao et al., 2022). Seven trials evaluated BCI-FES and nine evaluated BCI-Robot. No study directly compared the two BCI strategies. The overall evidence structure was therefore star-shaped, with conventional rehabilitation as the sole common comparator (Figure 2). In the main network, 259 participants were allocated to BCI-FES, 139 to BCI-Robot, and 390 to conventional rehabilitation. Detailed study characteristics are summarized in Table 1. Upper-limb recovery was the dominant outcome domain. The FMA-UE network included 12 studies, with six BCI-FES vs. conventional rehabilitation comparisons and six BCI-Robot vs. conventional rehabilitation comparisons (Supplementary Figure S1). The FMA-LE network was smaller and included four studies, comprising one BCI-FES comparison and three BCI-Robot comparisons (Supplementary Figure S2). In the stroke-stage subgroup analysis, 11 studies enrolled subacute populations and five enrolled chronic populations (Supplementary Figures S3 and S4).

**Figure 1:**
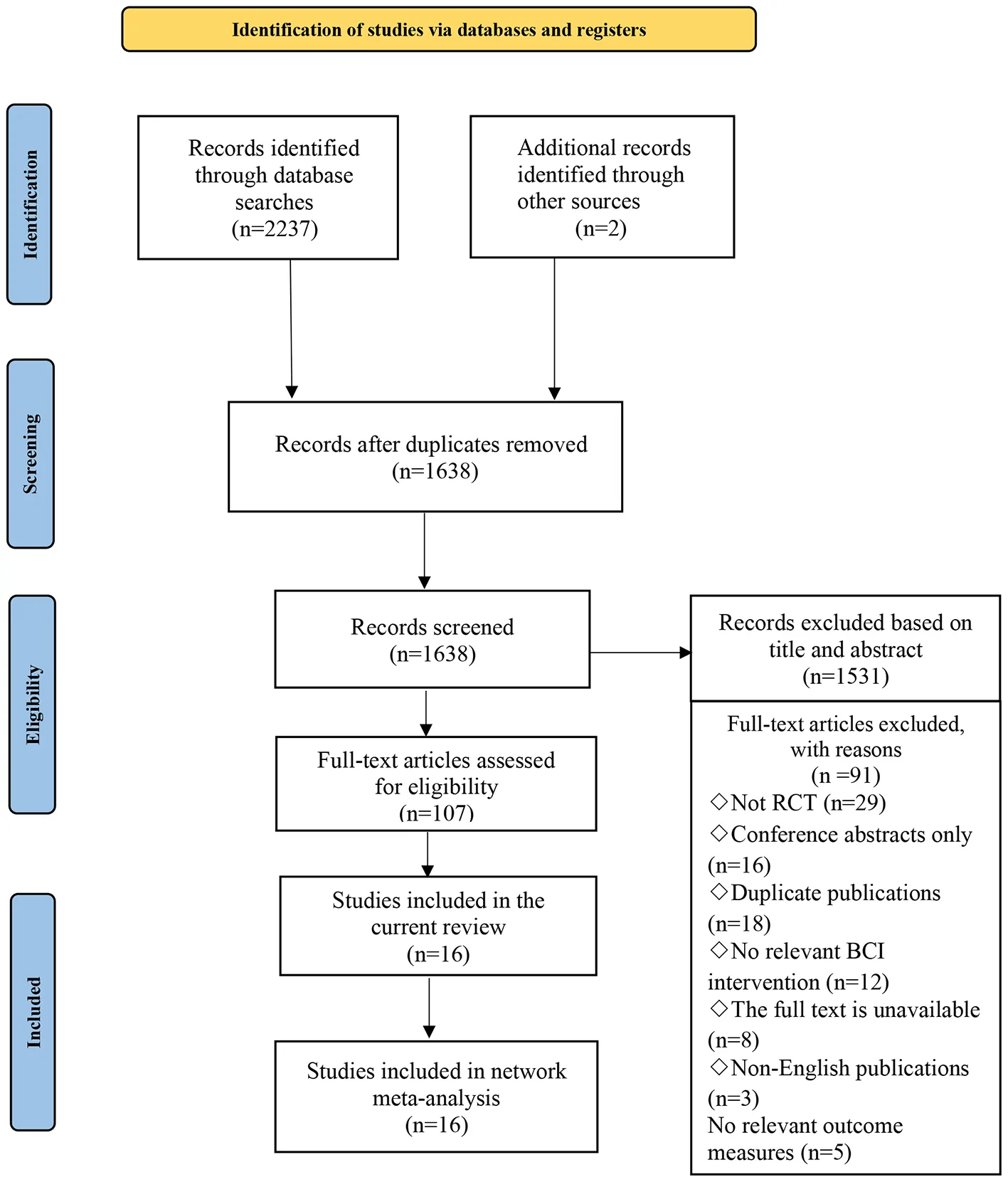
PRISMA flow diagram of the study selection process.

**Figure 2:**
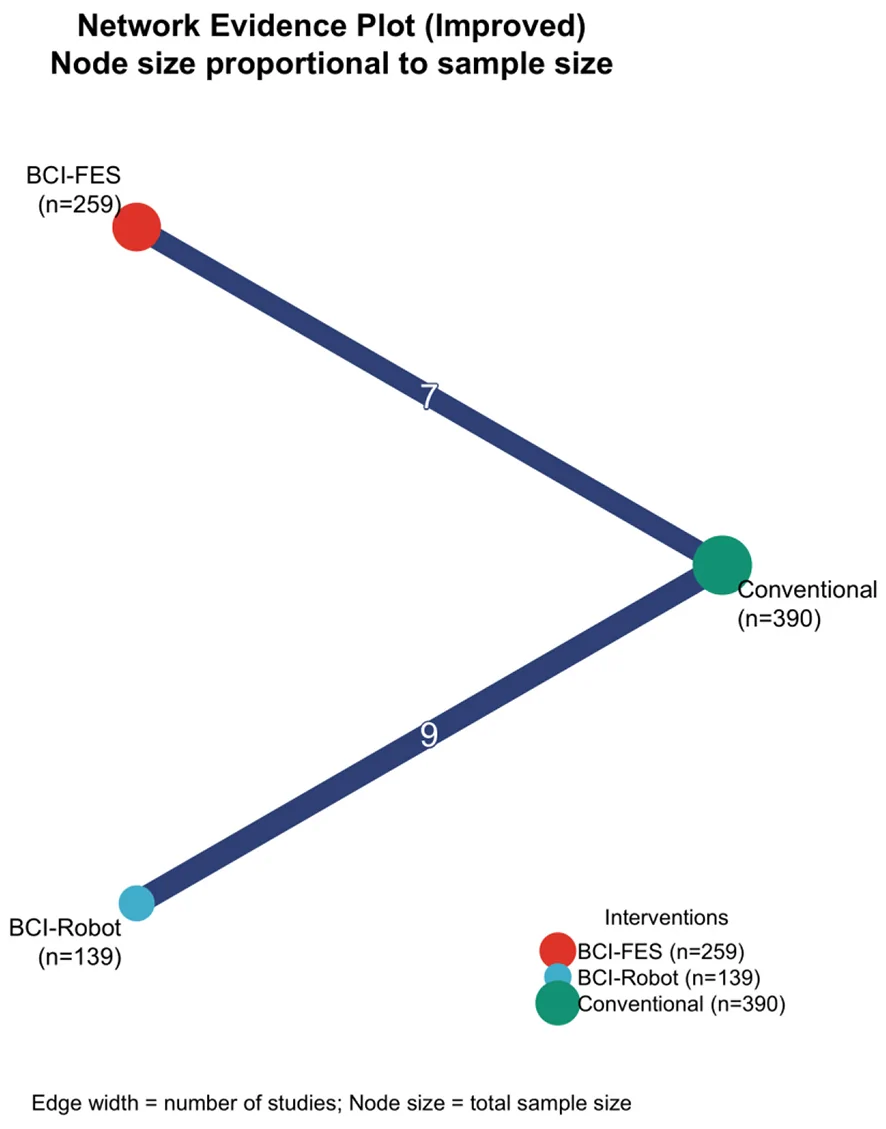
Overall network plot for the main comparison.

**Table 1:** Characteristics of included studies.

References	Domain	n (I/C)	Age, years (I/C), mean ±SD	Phase	Experimental intervention	Control	Training dose	Baseline motor score (I/C)	Outcomes
Chen et al. (2020)	UL	7/7	41.6 ± 12.0/52.0 ± 11.1	Subacute	BCI-hand exoskeleton + basic treatment	Motor imagery training	4 weeks; 3 sessions/week; 30 trials/set × 3 sets/session	31.3 ± 11.5/32.3 ± 11.8	①,⑪,⑫
Miao et al. (2020)	UL	8/8	48.8 ± 16.5/50.3 ± 16.8	Chronic	MI-BCI-FES + routine rehabilitation	Routine rehabilitation	4 weeks; 3 sessions/week; 2 runs/session, 60 trials/run	19.5 ± 9.9/20.6 ± 9.7	①,⑬,⑭
Hu et al. (2021)	UL	7/5	44.9 ± 7.5/60.4 ± 16.8	Chronic	MI-BCI multimodal feedback	Motor imagery training	6 weeks; 30 min/day; 7 days/week	12.7 ± 8.9/13.8 ± 6.7	①,②,③,④⑤
Li et al. (2021)	LL	12/13	51.2 ± 7.8/49.6 ± 8.4	Subacute	BCI lower-limb robot + routine rehabilitation	Routine rehabilitation	4 weeks; 30 min/day; 5 days/week	5.0 ± 2.0/5.4 ± 1.3	㉓,㉔,㉕
Yuan et al. (2021)	LL	16/14	61.8 ± 7.4/66.3 ± 5.9	Subacute	BCI pedaling system	Conventional pedaling	2 weeks; 24 sessions total; 2 sessions/day, 6 days/week	13.5 ± 8.8/13.7 ± 8.5	㉖
Zhao et al. (2022)	LL	14/14	50.1 ± 11.1/56.1 ± 11.5	Subacute	BCI robot + conventional physiotherapy	Sham BCI + conventional physiotherapy	4 weeks; 60 min/day; 6 days/week	10.4 ± 5.7/10.1 ± 6.8	㉓,㉔,㉘,㉙,㉚
Guo et al. (2022)	UL	10/10	60.2 ± 9.3/53.5 ± 8.3	Chronic	BCI robotic training + conventional therapy	Conventional therapy	2 weeks; 60 min/day; 5 days/week	14.9 ± 8.0/14.4 ± 8.7	①,⑥,⑦
Lee et al. (2022)	UL	13/13	55.2 ± 11.6/58.3 ± 9.2	Chronic	AOT + BCI-FES + conventional PT	FES + conventional PT	4 weeks; 30 min/day; 5 days/week	30.5 ± 5.1/29.5 ± 3.6	①,⑥,⑧,⑨,⑩,⑮
Li et al. (2022)	UL	12/12	43.8 ± 14.7/55.0 ± 12.2	Chronic	SMR-BCI multimodal feedback + rehabilitation	Conventional rehabilitation	2 weeks; 3 h/day; 5 days/week	22.6 ± 13.7/24.3 ± 17.1	①,⑥
Fu et al. (2023)	UL	30/31	55.9 ± 11.1/59.0 ± 14.5	Subacute	Portable BCI-hand exoskeleton + conventional therapy	Task-oriented guidance + conventional therapy	4 weeks; 30 min/day; 5 days/week	19.1 ± 11.1/23.3 ± 15.8	①
Ma et al. (2023)	UL	20/20	50.9 ± 12.6/58.3 ± 11.2	Subacute	MI-BCI robotic hand exoskeleton + conventional therapy	Conventional therapy	2 weeks; 40 min/day; 5 days/week	20.8 ± 11.2/20.8 ± 10.8	①,⑯
Liu et al. (2023)	UL	30/30	52.2 ± 11.8/51.2 ± 15.2	Subacute	MI-BCI-FES + conventional rehabilitation	Conventional rehabilitation + FES	3 weeks; 20 min/day; 5 days/week	31.6 ± 15.2/34.8 ± 13.8	①,⑥,④,⑰,⑱,⑲
Zanona et al. (2023)	UL	23/21	62.2 ± 9.8/61.0 ± 3.0	Subacute	BCI-hand exoskeleton + mental practice + OT	Occupational therapy	2 weeks; 5 sessions/week; 80 min/session	32.1 ± 16.8/37.0 ± 13.5	⑳,⑧,㉑,㉒,①
Wang et al. (2024)	UL	150/146	58.6 ± 10.9/59.1 ± 11.2	Subacute	EEG MI-BCI-FES + conventional rehabilitation	Conventional rehabilitation	4 weeks; 30 min/session; 5 days/week	23.9 ± 9.1/24.2 ± 9.4	①,③,⑧
Luo (2024)	LL	32/32	NR	Subacute	MI-BCI-FES + conventional rehabilitation	Conventional rehabilitation	2 weeks; 5 sessions/week; 60 min/day (2 × 30 min)	7.94 ± 1.24/8.06 ± 1.24	㉓,㉙,⑥
Ming et al. (2025)	UL	14/14	54.9 ± 8.7/60.8 ± 11.5	Subacute	KI-BCI + tDCS	tDCS alone	4 weeks; 30 min/session; 5 days/week	22.6 ± 3.3/22.3 ± 1.8	①,③,②,④

UL, upper limb; LL, lower limb; I/C, intervention/control; NR, not reported; BCI, brain-computer interface; FES, functional electrical stimulation; PT, physical therapy; OT, occupational therapy; tDCS, transcranial direct current stimulation; FMA-UE, Fugl-Meyer Assessment-Upper Extremity; FMA-LE, Fugl-Meyer Assessment-Lower Extremity; MBI, Modified Barthel Index.

Age and baseline motor scores are presented as mean ± SD. Stroke phase was classified according to the original studies. Circled numbers (–) in the “Outcomes” column correspond to the specific outcome measures listed in Supplementary Table S2.

### Risk of bias

The overall risk-of-bias profile is shown in Figure 3. Most studies were at low or unclear risk for random sequence generation and allocation concealment. Outcome assessment, incomplete outcome data, selective reporting, and other bias were rated as low risk in most trials. The main methodological limitation was performance bias, which is inevitable in device-based rehabilitation studies because blinding of participants and treating personnel is rarely feasible. A study-level summary of the risk-of-bias judgments is provided in Supplementary Figure S11.

**Figure 3:**
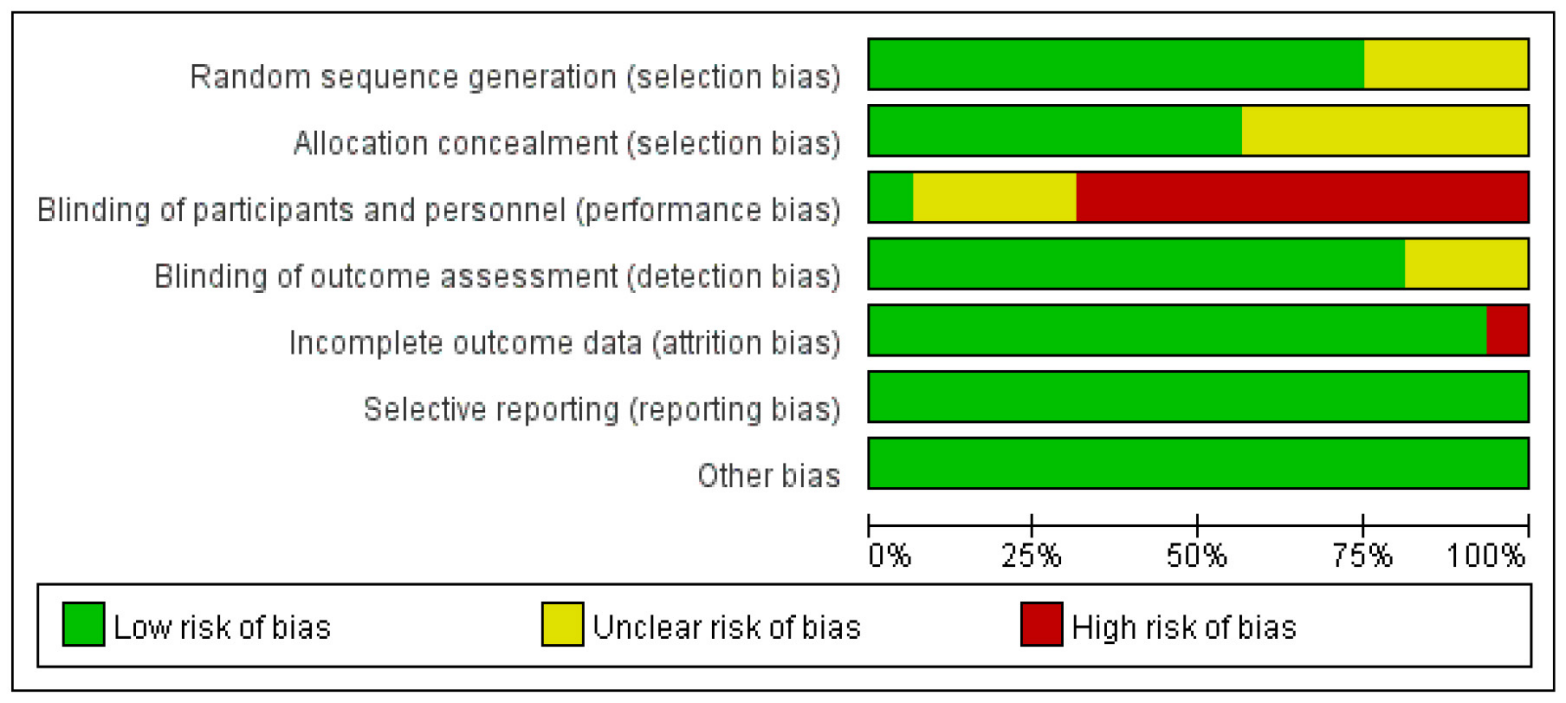
Risk-of-bias graph for included studies.

### Pairwise pooled effects by intervention type

Grouped random-effects meta-analysis showed an overall positive effect of BCI-based rehabilitation compared with conventional therapy (SMD = 0.36, 95% CI: 0.04–0.67; prediction interval: −0.82 to 1.53), with substantial heterogeneity (*I*^2^ = 69.5%, tau^2^ = 0.2773, *P* < 0.0001). The wide prediction interval indicates considerable uncertainty regarding the true effect in future settings. When stratified by intervention type, BCI-FES yielded a pooled effect of 0.57 (95% CI: 0.15–0.98; *I*^2^ = 73.6%), whereas BCI-Robot yielded a pooled effect of 0.17 (95% CI: −0.30 to 0.64; *I*^2^ = 67.3%). The test for subgroup differences was not significant (χ^2^ = 1.53, degrees of freedom = 1, *P* = 0.2155). These analyses are presented in Supplementary Figure S5 and should be regarded as supportive rather than definitive comparative evidence.

### Network meta-analysis

The main network estimates and treatment ranking are shown in Figure 4 and Table 2. Compared with conventional rehabilitation, BCI-FES was associated with a significant improvement in motor and functional recovery (SMD = 0.57, 95% CI: 0.12–1.01, *P* = 0.014). By contrast, the estimate for BCI-Robot vs. conventional rehabilitation did not reach statistical significance (SMD = 0.17, 95% CI: −0.25 to 0.60, *P* = 0.419). The indirect comparison between BCI-FES and BCI-Robot numerically favored BCI-FES, but the CI crossed zero (SMD = 0.39, 95% CI: −0.22 to 1.01), direct-indirect disagreement could not be evaluated. Because the network contained no closed loop, this comparison was informed by indirect evidence alone. A league-table heatmap is provided in Supplementary Figure S10 to summarize the direction and magnitude of the network estimates.

**Figure 4:**
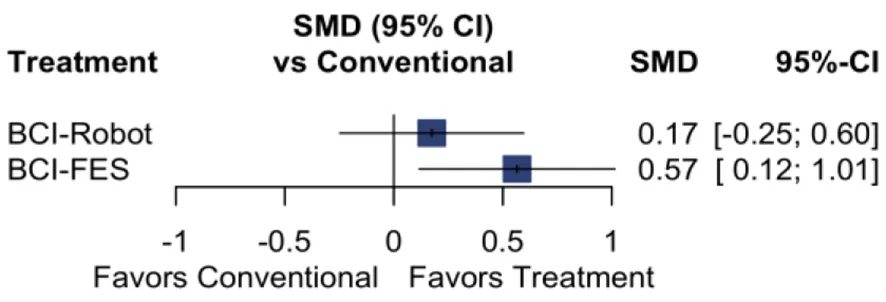
Main network estimates for motor and functional recovery after stroke.

**Table 2:** Main network meta-analysis results and treatment ranking.

Comparison	SMD (95% CI)	*P-value*	P-score (rank)
BCI-FES vs. conventional therapy	0.57 (0.12 to 1.01)	0.014	0.94 (1st)
BCI-Robot vs. conventional therapy	0.17 (−0.25 to 0.60)	0.419	0.45 (2nd)
BCI-FES vs. BCI-Robot	0.39 (−0.22 to 1.01)	NS	—
Conventional therapy	—	—	0.11 (3rd)

SMD, standardized mean difference; CI, confidence interval; NS, not statistically significant (confidence interval crosses the null). P-scores indicate the probability that a treatment ranks among the best.

### Treatment ranking

Treatment ranking was consistent with the pooled network estimates. BCI-FES ranked first with a P-score of 0.94, BCI-Robot ranked second with a P-score of 0.45, and conventional rehabilitation ranked last with a P-score of 0.11 (Table 2). BCI-FES had the highest P-score within the current network. Because formal inconsistency testing was impossible and some estimates were sensitive to influential trials, P-score rankings, including the first-place rank for BCI-FES, are purely exploratory. Ranking probabilities for all interventions are shown in Supplementary Figure S9.

### Subgroup networks

The subgroup networks indicated a broader and more balanced evidence base for upper-limb recovery than for lower-limb recovery. The FMA-UE network contained equal numbers of BCI-FES and BCI-Robot comparisons against conventional rehabilitation (Supplementary Figure S1), whereas the FMA-LE network was driven mainly by BCI-Robot studies (Supplementary Figure S2). Likewise, the subacute subgroup included more studies than the chronic subgroup, indicating that the current evidence is stronger in subacute stroke than in chronic stroke (Supplementary Figures S3 and S4).

### Small-study effects and sensitivity analyses

Visual inspection of the comparison-adjusted funnel plot did not suggest major asymmetry across the full network (Supplementary Figure S6). However, the number of studies per comparison was limited, so the assessment of small-study effects should be interpreted cautiously. Leave-one-out analyses showed that the BCI-FES estimate was influenced most strongly by the Luo 2024 trial (Luo, 2024). When this study was omitted, the pooled effect decreased from 0.57 to 0.29 and heterogeneity dropped from 73.6% to 0% (Supplementary Figure S7). For BCI-Robot, the Li 2021 trial (Li et al., 2021) had the largest influence. After omitting this study, the pooled estimate became statistically significant (SMD = 0.34, 95% CI: 0.03–0.66) and heterogeneity decreased from 67.3% to 32.7% (Supplementary Figure S8). The numerical treatment ranking was unchanged, but this did not resolve the influence of individual trials or strengthen the entirely indirect active-vs.-active comparison.

### Summary of findings

Taken together, the results suggest a more consistent signal for BCI-FES than for BCI-Robot when each was compared with conventional rehabilitation. The indirect active-vs.-active estimate was imprecise, and the certainty of evidence for this comparison was low to very low. The evidence for BCI-Robot was less stable, with wider confidence intervals and greater sensitivity to individual trials. Certainty ratings for the main comparisons are presented in Supplementary Table S1.

### Discussion

This network meta-analysis found that BCI-FES was associated with a clearer improvement in post-stroke motor recovery than conventional rehabilitation, whereas the estimate for BCI-Robot was positive but less precise. The indirect comparison between active output modalities numerically favored BCI-FES, but the confidence interval included no effect. These findings therefore suggest a difference in the consistency of available evidence rather than definitive superiority of one BCI modality over another.

One plausible explanation is that BCI-FES establishes a tighter intention-contingent sensorimotor loop. In these systems, cortical activity related to motor intention elicits peripheral electrical stimulation with negligible latency. This pairing of central activation and afferent input is consistent with associative plasticity models, which posit that repeated and precisely timed coupling may help strengthen residual motor pathways. Convergent evidence from paired associative stimulation and spike timing-dependent plasticity has shown that temporal precision is critical for shaping corticomotor excitability (Stefan et al., 2000; Wolters et al., 2003). In stroke rehabilitation, such timing-dependent mechanisms may account for why a BCI-FES system delivers a more biologically relevant training signal than systems in which feedback is less tightly linked to motor intention. Nevertheless, this mechanistic interpretation should be viewed as hypothesis-generating, because most included trials were not designed to test neurophysiological mechanisms directly.

Peripheral stimulation itself may contribute partially to the observed effect. Electrical stimulation is known to modulate corticomotor excitability, and its impact is strengthen when stimulation is contingent on motor intention rather than applied passively (Chipchase et al., 2011; Ridding and Taylor, 2001). This aligns with clinical work indicating that brain-actuated FES yields durable functional improvements and neurophysiological changes post-stroke (Biasiucci et al., 2018). Robotic systems, in contrast, primarily offer structured movement practice. Although they remain clinically valuable, their therapeutic effect is likely to depend on the extent of preserved voluntary effort and the fidelity with which the device is incorporated into a true closed-loop paradigm (Marchal-Crespo and Reinkensmeyer, 2009; Lo et al., 2010; Mehrholz et al., 2018).

The less consistent effect observed for BCI-Robot should not be interpreted as evidence of inefficacy. Robotic rehabilitation is an established way to deliver intensive and task-oriented practice post-stroke. However, robotic paradigms vary considerably in control strategy, assistance level, movement constraints, and the degree to which cortical intention is actually incorporated into the training loop (Marchal-Crespo and Reinkensmeyer, 2009; Mehrholz et al., 2018). Such variation is likely to increase between-study heterogeneity and dilute pooled estimates when diverse robotic systems are grouped under a single label. This interpretation is consistent with earlier reviews showing that design characteristics of BCI-based systems influence treatment response (Cervera et al., 2018; Mansour et al., 2022).

The more favorable signal observed in subacute stroke is also biologically plausible. Experimental and clinical evidence suggests that early post-stroke recovery represents a golden window of enhanced responsiveness to training, during which residual sensorimotor networks may be more amenable to reorganization (Zeiler and Krakauer, 2013). A closed-loop intervention that links intention to immediate peripheral reinforcement may therefore be particularly effective during this window. This subgroup finding, however, should not be overstated because the number of available studies was limited and definitions of stroke stage were not entirely uniform across trials.

Our findings refine the current understanding of BCI-based stroke rehabilitation. Earlier meta-analyses generally supported the efficacy of BCI-assisted therapy, and some suggested that combinations involving functional electrical stimulation might yield stronger effects than other feedback modalities (Bai et al., 2020; Cervera et al., 2018). By classifying BCI-FES and BCI-Robot as separate intervention nodes, the present analysis describes their clinical effect estimates relative to a common comparator. Although the categories differ in implementation, the outcome-level evidence does not demonstrate that the two approaches are mechanistically non-interchangeable or that one output modality causes superior recovery. That distinction matters, because not all BCI-based systems provide the same type or quality of closed-loop feedback.

These results also have practical implications for trial design. Future studies should move beyond broad labels such as “BCI-based rehabilitation” and report the engineering and therapeutic variables most likely to shape treatment response, including signal features, detection thresholds, system latency, feedback timing, assistance strategy, and co-interventions. The Stroke Recovery and Rehabilitation Roundtable has emphasized the need to align mechanisms, outcome measures, and biomarkers in stroke trials (Boyd et al., 2017). In this field, adequately powered head-to-head comparisons of BCI-FES and BCI-Robot, with prespecified stratification by stroke stage and impairment severity, would be especially informative.

Several limitations should be considered. The network was star-shaped and contained no direct comparisons between the two active output modalities, so the active-vs.-active estimate was entirely indirect. The included trials were small and heterogeneous in BCI system design, treatment dose, target limb, stroke stage, and comparator intervention. Blinding of participants and therapists was generally not feasible, which may have increased performance bias. Some estimates were sensitive to individual trials, and long-term follow-up data were limited. The absence of closed loops also precluded formal inconsistency testing; thus, the consistency assumption could not be checked against the data. Restricting the review to studies published from 2020 onward may have omitted relevant earlier trials and affected the precision, network structure, and effect estimates. Our conclusions are therefore limited to the specified publication period. These limitations reduce confidence in treatment rankings and prevent firm clinical recommendations about the preferred BCI output modality.

Despite these limitations, the present analysis helps shift the focus of the field from whether BCI-based rehabilitation is beneficial in general to which closed-loop design features are most relevant to clinical recovery. The available evidence suggests that timely, intention-contingent sensory feedback may be an important therapeutic ingredient, although this remains to be tested directly. That interpretation is consistent with current neurophysiology, prior BCI literature, and broader rehabilitation principles that emphasize active and task-relevant practice (Biasiucci et al., 2018; Stefan et al., 2000; Zeiler and Krakauer, 2013).

Future trials should directly compare BCI-FES with BCI-Robot using standardized protocols and clearer reporting of engineering variables. Longer follow-up and incorporation of neurophysiological biomarkers alongside clinical outcomes will also be important for identifying which patients benefit most and for clarifying the mechanisms that support recovery (Baniqued et al., 2021; Boyd et al., 2017).

## Conclusion

In conclusion, BCI-assisted rehabilitation appears to be a promising adjunct for post-stroke motor recovery. Within the current evidence network, BCI-FES showed a more consistent signal of benefit than BCI-Robot, but this finding was based on indirect evidence and should not be interpreted as proof of comparative superiority. Direct comparative trials with standardized intervention reporting, clinically meaningful outcomes and longer follow-up are needed to guide the selection of BCI output modalities in stroke rehabilitation.

## Data Availability

All data analyzed in this study are included in the article and its Supplementary material.
