# Supplementary material for: Effects of brain–computer interface output modalities on motor recovery after stroke: a systematic review and network meta-analysis
Source: Front Neurosci. 2026 Oct 27;20:1978309. doi: 10.3389/fnins.2026.1978309 (PMC13748378; doi:10.3389/fnins.2026.1978309)
Supplement: Supplementary file 1 [file Data_Sheet_1.docx]

**Supplementary Table S1** CINeMA-based certainty assessment for the main network comparisons

| Comparison | Outcome | Evidence source | No. of studies | Within-study bias | Reporting bias | Indirectness | Imprecision | Heterogeneity | Incoherence | Confidence |
| --- | --- | --- | --- | --- | --- | --- | --- | --- | --- | --- |
| BCI-FES vs conventional therapy | Motor function (FMA) | Mostly direct | 7 | Some concerns | No concerns | No concerns | No concerns | Some concerns | No important concerns* | ⊕⊕⊕◯ Moderate |
| BCI-Robot vs conventional therapy | Motor function (FMA) | Mostly direct | 9 | Some concerns | No concerns | No concerns | Some concerns | Some concerns | No important concerns* | ⊕⊕◯◯ Low |
| BCI-FES vs BCI-Robot | Motor function (FMA) | Indirect/ network estimate | 16 | Some concerns | No concerns | Major concerns | Some concerns | Some concerns | Some concerns* | ⊕◯◯◯ Very low |

Notes: Use the wording in the incoherence column only if the local and global inconsistency assessments did not show important incoherence. If inconsistency was detected, the incoherence judgment should be revised accordingly. For the indirect comparison (BCI-FES vs BCI-Robot), the number of studies refers to the total studies informing the network estimate. *Incoherence was not formally testable because the network contained no closed loops. Confidence ratings are presented using the GRADE-style symbol format: ⊕⊕⊕⊕ high, ⊕⊕⊕◯ moderate, ⊕⊕◯◯ low, and ⊕◯◯◯ very low.

Abbreviations: BCI, brain-computer interface; FES, functional electrical stimulation; FMA, Fugl-Meyer Assessment; CINeMA, Confidence in Network Meta-Analysis.

**Supplementary Table S2.** Mapping of outcome codes to specific measures

（1） FMA-UE (Fugl-Meyer Assessment for Upper Extremity)
（2） ARAT (Action Research Arm Test)
（3） MSS (Motor Status Scale)
（4） MBI (Modified Barthel Index)
（5） sEMG (surface electromyography)
（6） WMFT (Wolf Motor Function Test)
（7） MAS (Modified Ashworth Scale)
（8） MAL (Motor Activity Log)
（9） MAL-AOM (MAL – Amount of Use subscale)
（10） MAL-QOM (MAL – Quality of Movement subscale)
（11） ERD (event-related desynchronization)
（12） Topographic changes (EEG topography)
（13） BCI classification accuracy
（14） EEG patterns (including PSD/CSP topographies)
（15） IADL (Instrumental Activities of Daily Living)
（16） rs-fMRI (resting-state functional magnetic resonance imaging)
（17） ANT (Attention Network Test)
（18） SDMT (Symbol Digit Modalities Test)
（19） Schulte Grid Test
（20） FIM (Functional Independence Measure)
（21） BBT (Box and Blocks Test)
（22） JHFT (Jebsen Hand Function Test)
（23） FMA-LE (Fugl-Meyer Assessment for Lower Extremity)
（24） FMA-B (Fugl-Meyer Assessment for Balance)
（25） Holden Walking Function Assessment
（26） FMA-L (Fugl-Meyer Assessment for Lower Limb)
（27） FAC (Functional Ambulation Category)
（28） Serum BDNF levels
（29） MEP (motor-evoked potential)
（30） DTI/DTT (diffusion tensor imaging / tractography)

Note: Numbers in parentheses (1 to 30) correspond to the circled numbers (① to ㉚) used in Table 1 of the main text.

Supplementary Fig. S1 Network plot for the upper-limb motor outcome (FMA-UE)

Supplementary Fig. S2 Network plot for the lower-limb motor outcome (FMA-LE)

Supplementary Fig. S3 Network plot for studies in the subacute stroke stage

Supplementary Fig. S4 Network plot for studies in the chronic stroke stage

**Supplementary Fig. S5** Grouped pairwise forest plot by intervention type

**Supplementary Fig. S6** Comparison-adjusted funnel plot

**Supplementary Fig. S7** Leave-one-out sensitivity analysis for BCI-FES

**Supplementary Fig. S8** Leave-one-out sensitivity analysis for BCI-Robot

Supplementary Fig. S9 Rankogram of treatment ranking probabilities

Supplementary Fig. S10 League table heatmap of pairwise comparisons

**Supplementary Fig. S11** Study-level risk-of-bias summary
